# Humanizing Big Data: Recognizing the Human Aspect of Big Data

**DOI:** 10.3389/fonc.2020.00186

**Published:** 2020-03-13

**Authors:** Kathy Helzlsouer, Daoud Meerzaman, Stephen Taplin, Barbara K. Dunn

**Affiliations:** ^1^Division of Cancer Control and Population Sciences, National Cancer Institute, Bethesda, MD, United States; ^2^Center for Biomedical Informatics and Information Technology, National Cancer Institute, Bethesda, MD, United States; ^3^Center for Global Health, National Cancer Institute, Bethesda, MD, United States; ^4^Division of Cancer Prevention, National Cancer Institute, Bethesda, MD, United States

**Keywords:** big data, predictive analytics, precision medicine, cancer risk prediction, clinical genetics/genomics, direct-to-consumer testing, data sharing

## Abstract

The term “big data” refers broadly to large volumes of data, often gathered from several sources, that are then analyzed, for example, for predictive analytics. Combining and mining genetic data from varied sources including clinical genetic testing, for example, electronic health records, what might be termed as “recreational” genetic testing such as ancestry testing, as well as research studies, provide one type of “big data.” Challenges and cautions in analyzing big data include recognizing the lack of systematic collection of the source data, the variety of assay technologies used, the potential variation in classification and interpretation of genetic variants. While advanced technologies such as microarrays and, more recently, next-generation sequencing, that enable testing an individual's DNA for thousands of genes and variants simultaneously are briefly discussed, attention is focused more closely on challenges to analysis of the massive data generated by these genomic technologies. The main theme of this review is to evaluate challenges associated with big data in general and specifically to bring the sophisticated technology of genetic/genomic testing down to the individual level, keeping in mind the human aspect of the data source and considering where the impact of the data will be translated and applied. Considerations in this “humanizing” process include providing adequate counseling and consent for genetic testing in all settings, as well as understanding the strengths and limitations of assays and their interpretation.

## Introduction

Precision medicine in cancer treatment is defined by the National Cancer Institute as a “genetic understanding” of cancer, offering a specific treatment tailored to an individual (1). Cancer results from a variety of factors, both genetic and environmental. The developmental path to the actual tumor results from an accumulation of genetic changes which vary across and within tumors. Some of these genetic changes are inherited germline mutations, but the majority are somatic changes, uncorrected by DNA repair processes, that result from exposures or random events. These genetic changes may present treatment targets; however, the genetic changes are heterogeneous and specific actionable treatment targets may be rare. To detect these changes, data on many tumors in many patients are required. Similarly, germline genetic changes that are inherited may increase susceptibility to cancer either directly by affecting key proteins such as those critical to repairing DNA damage or by increasing susceptibility to effects of cancer-causing environmental factors. These germline changes may also be very rare; thus, analysis of large datasets is required to determine if there is an association with cancer development and to determine if the changes are useful in predicting risk.

The search for treatment targets and for predictive analytics has fueled the demand for large data sets, i.e., “big data.” Despite the current widespread use of the term, no consistent or single definition of “big data” has been agreed on (2–5). The online Oxford Dictionaries definition is: “extremely large data sets that may be *analyzed computationally* to reveal patterns, trends, and associations, especially relating to human behavior and interactions” (6). In essence, “big data” denotes any data set large enough to permit valid use of *statistically based analytical methods* to extract a level of knowledge in an area of interest.

This massive data collection requires combining data from varied sources, collected in disparate manners and assayed using multiple techniques. The specific application of big data to be discussed in this paper is genomics and related omics as they feed into clinical management of patients.

These large data sets can be extremely complex, typically characterized by references to the “Vs” [high volume, velocity, variety, veracity, value, variability (4, 5, 7, 8)]. The growth in acquiring and using “big data” is due to a variety of factors including an increase in research and clinical applications of genetic findings, pharmaceutical company interest in large datasets to develop and apply targeted treatments, consumer interest in genetic tests for ancestry and medical applications, and a growth in the direct-to-consumer genetic test market. “Big Data” is now big business and growing. The market for genetic testing is projected to exceed $22 billion by 2024 (9). Companies now produce, buy, and sell genetic data. Buyers of data include researchers and pharmaceutical companies. Sellers include companies that provide genetic testing and/or companies that build and sell access to large data sets (*data aggregators*), as well as a new developing market for individuals, not just the companies, to benefit monetarily from the selling of their data to companies (10). Companies that market DNA data also may offer to perform testing. With this developing business around producing and sharing data, outside of the clinical setting, the danger exists of losing site not only of both how the data were collected and assayed, but also of the individual who is sharing the most intimate of data, their genetic profile.

The sharing and aggregation of genetic information into large data sets may obscure the fact that the basic underlying source of each data point is an individual. Individuals provide the data, the data from many are aggregated, and ultimately the information is translated back to an individual. Thus, analyzing and interpreting big data require recognizing the individual source of the data, how the data are obtained, stored, and assayed and analyzed, and how, ultimately, to apply them. In essence, the data must always be viewed and used with the humanity of the individuals providing their genetic material kept in mind.

The main theme of this review is to discuss challenges associated with big data in general and specifically to bring the sophisticated technology of genetic/genomic testing down to the individual level, where the impact of the data will be translated and applied. The latter activities reflect the “humanizing” of big data as applied to genomic medicine. This article will address analytical aspects of both genetics and genomics data and their evolution over time. Whereas, “genetics” involves the functioning and make-up of individual genes, the field addressed by big data sets containing genetic information is “genomics”: genomics deals with *all* genes in an organism and their inter-relationships (11). The additional complexity in such big data has downstream implications for clinical interpretation and management for the individual. For this article, we will use the term “genetics” to include both genetics and genomics data, and we will address primarily germline genetics (i.e., also genomics), as elaborated below. Finally, as we review the sequential stages of genetic testing, we wish to re-emphasize the need to consider the relationship of each technical phase of the pipeline to the human being who is the source of the genetic material being analyzed.

## Challenges to Analysis of Genomic and Medical Data for Discovery of Clinically Relevant Genetic Variants

### Laboratory Testing of Germline DNA Variants

Testing of the germline for DNA variants, passed from one generation to the next, that confer deleterious phenotypic attributes has evolved radically over the years. This is largely in response to the evolution of technologies that enable massive testing of the genome (12), including microarrays but especially next-generation sequencing (13, 14). The huge data sets generated by these methods pose major challenges to the next stage in the pipeline: bioinformatic analysis and statistical validation. Such laboratory technologies allied with their follow-up bioinformatic analyses provide the venue through which “big data” are generated, and then funneled down into clinically interpretable genetic information, i.e., that which is directly relevant to the patient.

Challenges to analyzing genomic data for knowledge discovery begin in the laboratory at the technical level in the choice and conduct of specific approaches to sample preparation and laboratory analysis (15). The challenges continue downstream with the initial phases of the bioinformatic pipeline for identification of clinically relevant variants. These initial challenges involve selection of algorithms for optimal filtering of genetic variants and are followed down the pipeline through selection of appropriate algorithms at all subsequent informatic stages necessary to identify meaningful variants (15). Furthermore, the very large number of loci interrogated in such discovery research represent individual tests for clinically relevant genetic variants, posing the statistical challenge inherent in multiple testing and concerns about identifying false positives. The quality of the data generated at the end of this genomic pipeline, i.e., the data on which clinical associations will be based, must be carefully monitored throughout. Bias and variable thresholds for calling individual genetic variants as clinically relevant can feed into erroneous conclusions drawn from data. Scrutiny of the findings at each stage of the pipeline is essential to maximize the chance of identifying true positive variants and avoid missing false negatives. Furthermore, impediments to generation of accurate, meaningful data are not limited to technical decisions but are subject as well to inconsistent communications among researchers with differing expertise at each stage of the genomic pipeline (15). Cautionary approaches are therefore necessary if the users of the genomic findings in the healthcare setting can trust the quality of the underlying data.

### Addressing the Limitations of Genomic Technologies: Analytic Validity and Probabilistic Outcomes

The laboratory technologies allied with their follow-up bioinformatic analyses provide the venue through which “big data” are generated, and then funneled down into clinically interpretable genetic information, where “humanization” of the “big data” needs to be emphasized. This stage is where the “variety” attribute of generated data must be sifted through to glean out irrelevant findings and select for meaningful outcomes that are potentially pertinent to clinical interpretation. Key to humanizing the data is communicating to the patient the limitations at the clinical level of the transmitted information, both technical and genetic.

The platforms most commonly used to identify pathogenic variants in the clinical setting are single nucleotide polymorphism (SNP) chip (microarray)-based and next generation sequencing (next gen sequencing)-based technologies. Although they are used in standard clinical practice, caution must be exercised in interpreting the results of these analytic tools. They are not perfect, and the limitations of the diagnostic accuracy, or analytic validity (16), of a given platform must be considered when communicating results to a patient. This is particularly true of SNP chips. When juxtaposed against results obtained from next gen sequencing, the diagnostic accuracy of SNP chips has been shown to be uncertain when used to detect rare pathogenic variants in the general population (17). The analytic validity of such rare variants is poor, leading to a very high false discovery rate. Thus, although SNP chips are useful for assessing the presence of common variants in a given population, such as polymorphisms, this does not translate into the rare variants relevant to clinical genetic diagnoses. Similar limitations exist for SNP chips from different manufacturers. This contrasts with sequencing platforms which are not affected by the same technical issues as chips and are therefore more accurate in genotyping rare variants (17).

Even in a setting of strong analytic validity, as seen with sequencing, many uncertainties remain. An accurately identified variant may have questionable clinical validity, the strength of its association with the phenotypic outcome of interest (16, 18), i.e., disease, being uncertain. These unknowns are inherent in the probabilistic nature of phenotypic expression of genetic variants. Patients may assume that identification of a pathogenic variant equates to certain development of the associated disease, whereas incomplete penetrance is generally the rule in heritable diseases such as adult cancers. Nevertheless, the actual penetrance of rare alleles is uncertain and can be over-estimated by clinical ascertainment methods (19). Even greater uncertainty exists for variants with unknown pathogenicity, namely “variants of uncertain significance,” or VUSs. Without humanizing such findings by communicating the absence of documented clinical relevance to the patient, unnecessary anxiety may be provoked and avoidable invasive treatment interventions undertaken. Finally, documentation of analytic and clinical validity is not sufficient to make a genetic test truly useful to the patient. The test must have clinical utility in that it lays the groundwork for beneficial interventions, whether pharmaceutical, surgical, or behavioral, without overriding risks (16). By establishing that a genetic test can lead to a clinically actionable intervention, the role played by big data in performance of the test becomes humanized.

## Challenges to Management of Big Data: Genomic and Clinical Data

### Ethical Challenges

Ethical issues evolving from the amassing of genetic data should be addressed by researchers, health care providers and companies. Subsequent use of “big data” must consider the selective nature of the source of the data, i.e., the patient, and the generalizability as well as the absolute necessity to prevent data breaches and ensure data security (8). Informed consent is an essential part of this process. The sharing of information from big data accumulated from thousands of individuals, has long raised concerns about maintaining individual privacy while advancing our understanding of genetic associations that will promote public health (8, 20, 21). The potential disregard of maintaining genetic privacy has led to anxiety about sequelae involving discrimination in multiple aspects of life, including employment and health insurance (20). While the Genetic Information Nondiscrimination Act (GINA) was enacted to prohibit such discriminatory behavior, additional domains (e.g., life, disability, and long-term care insurance) have remained vulnerable to misuse of genetic information (20). The ethical issues arising from the need to optimize these two “goods”– health vs. privacy—while balancing the risks and benefits emerging from this process (22) constitute an essential part of humanizing the big data.

### Security Challenges

Although security challenges overlap those inherent in the ethical concerns just described, a number of issues relating to security merit independent mention. Data needs to be accessible and at the same time secure. Security must guarantee privacy of data relating to the individual. An actual set of criteria, FISMA (Federal Information Security Management Act), provides a framework to guide protections of any information involving government activities. The private sector parallel is HIPAA (Health Insurance Portability and Accountability Act), which is widely adhered to in healthcare settings. These security concerns are becoming increasingly challenging due to the explosion of big data and their storage on multiple cloud resources (23).

### Challenges to Management of Data Size and Data Storage (the Silo Problem)

The huge size of big data, exacerbated by its continuous growth in volume, poses challenges to storage (5, 24). Traditionally data have been generated and stored in isolated compartments that may even differ qualitatively from each other. As an example, different departments in the same organization may store data in their own data bases, resulting in “data silos.” The content of siloed data in different departments may overlap but be encoded using differing terminology such that these data cannot “speak to each other.” This creates a serious impediment to integrated analyses of healthcare-related data across siloes; such analyses are critical to understanding factors affecting health-directed outcomes, including genetics. Among critical siloed data sets are Electronic Health Records (EHRs) (23), valuable for generating trends and predictive models, including genomic and pharmacogenomic markers (5, 25). The huge size of certain types of data, i.e., genomic data, which must be integrated with other data types of smaller size but much greater complexity, i.e., phenotypic data as contained in the EHR, poses additional challenges, which will be discussed below.

### Challenges to Management of Data in Unstructured Formats (26)

Frequently superimposed on the sheer size and ongoing growth of the data is the extreme architectural complexity of the data. The complexity of certain types of data (e.g., genomic) poses daunting challenges to being moved from home storage to an analytic environment. Unstructured data does not conform to a consistent accessible framework and language. Therefore, it needs to be converted into a structured readable format in order to identify useful information. In the clinical genomic setting, this conversion to a structured format is essential to teasing out genetic variants that are clinically meaningful and actionable. Historically, medical charting was entirely unstructured, comprising handwritten notes interspersed with machine-generated data, such as laboratory values. The EHR represents a first step at structuring such patient data by providing a consistent template for entries of medical information (23). However, data derived from the EHR are of multiple types (27). One estimate has 80% of data contained in EHRs as unstructured (26, 28). These varied entries in the EHR have value in that they can be used to formulate phenotypic classifications of patients. The technical challenges to this conversion process involve sophisticated algorithms using machine learning, natural language processing (NLP), and artificial intelligence (AI) (26). In the clinical genetic setting, examples of unstructured data that are difficult to convert to structured formats include EHRs, genomics, and other omic datasets. Commonly, for example, integration of the EHR with genomic and other types (e.g., biospecimen) of clinically relevant data results in questionable phenotypic diagnoses due to inaccurately determined correlations (29, 30). In essence, challenges to data quality, reliability, accuracy and integration must always be addressed. The ultimate goal is to discover associations between genetic/genomic variations and clinical phenotypes that are accurate and clinically meaningful in that they can be used to manage patient care, essentially creating predictive models (26).

### Challenges to Data Sharing

Essential to gleaning meaningful, actionable information from large data sets, in any context, is sharing of data among data producers (31). Given the need for as much data as possible to deduce clinically meaningful genomic variants, sharing of data among source clinical sites is critical, especially for rare genetic diseases (32). A guideline known as FAIR (Findable, Accessible, Interoperable, and Reusable) has been developed to guide investigators in managing the sharing of big data (33). To optimize the quality and usefulness of shared data sets, regulatory policies governing all genomic-related data generated by NIH-funded research have been established. Such Genomic Data Sharing (GDS) policies are specific to given types of data (34).

### Challenges to Testing of the Individual, i.e., the Data Source

Sources for large analytic data sets, i.e., “big data,” include data from clinical settings as well as genetic testing companies. Thus, potential selection factors for who gets testing will affect the results and interpretation. Until recently, the ordering of cancer genetic tests for cancer susceptibility syndromes for those diagnosed with cancer or with a strong family history of cancer was done in the clinical setting, after genetic counseling by a qualified health care provider. More recently, cancer genetic testing, as well as other health-related genetic testing, has expanded beyond the clinical setting, with companies advertising and offering testing directly to consumers without the need for involving a health care provider, or offering the test with a company-provided physician to order the test. The benefit of direct-to-consumer testing is potentially improved accessibility through convenience of in-home testing, bypassing requirements for health care provider visits, and lower cost tests. Data sets with a preponderance of clinically sourced data are likely to have higher risk individuals than direct-to-consumer or consumer-driven genetic testing. Also, in contrast to direct-to-consumer generated data, clinical settings are more likely to have extensive family history information, which is critical for interpreting test results. However, the extensive family history documentation may or may not be adequately or accurately transmitted to the “big data” compilation.

The individual who is the source of the data, the researcher analyzing it, and the clinicians who use the results of analyses should have a broad understanding of the process of consent, genetic testing, its benefits, harms and limitations, the potential implications of data sharing and with whom genetic testing results are shared. Immersed in the massive amounts of information and issues surrounding the use of genetic/genomic data at the clinical level, the input source of these data—the patient/individual—and the process of generating the data may be overlooked.

Pre-test counseling prior to proceeding with genetic testing is recommended because of the complexity of genetic information, and the need to anticipate how that information will be used for subsequent management of risk. Counseling includes several key components: medical and family history, risk assessment, risk perception, discussion of the most appropriate test, benefits and limitations of testing, communication with family members, and follow-up management (35, 36). This patient-centered approach espouses shared decision making, a process by which the patient has an informed discussion with the health care provider about the above issues, taking into consideration their personal values and whether or not to pursue genetic testing. Pre-test genetic counseling informs the individual and facilitates shared decision making while ensuring patient autonomy in the process (37) and is recommended by the U. S. Preventive Services Task Force (USPSTF) (38) and the National Comprehensive Cancer Network (NCCN) (39) in appropriate situations. Unlike other medical tests, genetic testing has implications for the family members, leading to issues such as how to communicate test results to family members as well as how the data may be shared. These downstream components of the genetic pipeline illustrate the strong human element with which the process culminates. Those using big data should ensure that the individual's preferences are respected and that they are informed of the potential broad sharing of data. Similarly, when applying information gathered from analyses of “Big Data,” the uncertainty that may be introduced by the methodologic issues in data generating activities as noted previously should be considered. Progress in technical and computational methodologies has simplified the generation of massive genomic analyses but limitations still exist.

## Summary

The application of technologies to generate and interpret big data related to genetic testing holds promise for the future of cancer medicine. The practice of “precision medicine,” in which the diagnostic and therapeutic interactions are tailored to a given patient, should benefit considerably from modern genomic technologies. Unquestionably genetic understanding is a key component of this approach to patient care, given the foundational role played by cumulative somatic mutations in carcinogenesis (40). Precision medicine must be built on precision data. The sources of the data used in “big data” should be stated along with the characterization of the population source, specimen source and preparation, assays used and analytic methods and algorithms employed. At the application and interpretation of data, the “precision” of precision medicine derives as much from an understanding of the psychological and social setting and needs of the patient and from the standard clinical attributes that brought the individual to the medical system as from the genetic underpinnings of the cancer or cancer risk. The composite of all these attributes makes the focus on a given patient truly precise, humanizing the process of incorporating genetic content into the practice of cancer medicine.

The potential of technology to improve the public health is unquestionable. However, understanding how technical platforms that analyze large-scale data feed into clinically relevant information can be daunting for patients and healthcare providers without specific genomic training. In this paper we have drawn attention to the many challenges and limitations as well as benefits associated with analyzing and applying big data to clinical applications. Our goal has been to point the way to demystifying the complexity of “big data” so that recipients of its benefits, patients and providers, will be in a better position to make appropriate clinical decisions. In this sense, we have attempted to “humanize big data,” by unraveling its many components in an effort to make its meaning, if not all its details, more accessible to non-specialists.

## Author Contributions

All authors listed have made a substantial, direct and intellectual contribution to the work, and approved it for publication. KH, BD, and DM contributed most of the writing.

### Conflict of Interest

The authors declare that the research was conducted in the absence of any commercial or financial relationships that could be construed as a potential conflict of interest.

## References

[1] Precision Medicine in Cancer Treatment 2019. Available online at: https://www.cancer.gov/about-cancer/treatment/types/precision-medicine (accessed October 3, 2017).

[2] Su P. Direct-to-consumer genetic testing: a comprehensive view. Yale J Biol Med. (2013) 86:359–65. 24058310 PMC3767220

[3] Wikipedia. Big Data 2019. Available online at: https://en.wikipedia.org/wiki/Big_data; https://webcache.googleusercontent.com/search?q=cache:ISElu6k9ARwJe; https://en.wikipedia.org/wiki/Big__data+&cd=1&hl=en&ct=clnk&gl=us (accessed February 25, 2019).

[4] Na KS Han C Kim YK. Big data and discovery sciences in psychiatry. Adv Exp Med Biol. (2019) 1192:3–15. 10.1007/978-981-32-9721-0_1 31705487

[5] Andreu-Perez J Poon CC Merrifield RD Wong ST Yang GZ. Big data for health. IEEE J Biomed Health Inform. (2015) 19:1193–208. 10.1109/JBHI.2015.2450362 26173222

[6] Dictionaries O Oxford Dictionaries. Oxford: Lexico.com (2019). Available online at: https://www.lexico.com/en/definition/big_data

[7] Fuller D Buote R Stanley K. A glossary for big data in population and public health: discussion and commentary on terminology and research methods. J Epidemiol Community Health. (2017) 71:1113–7. 10.1136/jech-2017-209608 28918390

[8] Herschel R Miori VM Ethics & big data. Technol Soc. (2017) 49:31–6. 10.1016/j.techsoc.2017.03.003

[9] Genetic Testing Market Surge to Cross $22 Billion By 2024: MarketWatch. (2019). Available online at: https://www.marketwatch.com/press-release/genetic-testing-market-will-register-116-growth-to-cross-usd-225-billion-by-2024–2019-02–26 (accessed February 22, 2019).

[10] Zhang S Big Pharma Would Like Your dna 23 and meś $300 Million Deal With Glaxosmithkline is Just the Tip of the Iceberg. The Atlantic (2018).

[11] Glossary of genomics terms. JAMA. (2013)309:1533–5. 10.1001/jama.2013.2950

[12] Meerzaman D Dunn BK Lee M Chen Q Yan C Ross S. The promise of omics-based approaches to cancer prevention. Semin Oncol. (2016) 43:36–48. 10.1053/j.seminoncol.2015.09.004 26970123

[13] Metzker ML. Sequencing technologies - the next generation. Nat Rev Genet. (2010) 11:31–46. 10.1038/nrg2626 19997069

[14] Adams DR Eng CM. Next-generation sequencing to diagnose suspected genetic disorders. N Engl J Med. (2018) 379:1353–62. 10.1056/NEJMra1711801 30281996

[15] Meerzaman D Dunn BK. Value of collaboration among multi-domain experts in analysis of high-throughput genomics data. Cancer Res. (2019) 79:5140–5. 10.1158/0008-5472.CAN-19-0769 31337654 PMC6801074

[16] Burke W. Genetic tests: clinical validity and clinical utility. Curr Protoc Hum Genet. (2014) 81:9.15.1–8. 10.1002/0471142905.hg0915s81 24763995 PMC4084965

[17] Weedon M Jackson L Harrison J Ruth K Hattersley A Wright C Very Rare Pathogenic Genetic Variants Detected by SNP-Chips Are Usually False Positives: Implications for Direct-to-Consumer Genetic Testing Online. Cold Spring Harbor Laboratory (2019).

[18] Zion TN Wayburn B Darabi S Lamb Thrush D Smith ED Johnston T. Clinical validity assessment of genes for inclusion in multi-gene panel testing: a systematic approach. Mol Genet Genomic Med. (2019) 7:e630. 10.1002/mgg3.630 30900393 PMC6503028

[19] Wright CF West B Tuke M Jones SE Patel K Laver TW. Assessing the pathogenicity, penetrance, and expressivity of putative disease-causing variants in a population setting. Am J Hum Genet. (2019) 104:275–86. 10.1101/407981 30665703 PMC6369448

[20] Green RC Lautenbach D McGuire AL. GINA, genetic discrimination, and genomic medicine. N Engl J Med. (2015) 372:397–9. 10.1056/NEJMp1404776 25629736

[21] Salerno J Knoppers BM Lee LM Hlaing WM Goodman KW. Ethics, big data and computing in epidemiology and public health. Ann Epidemiol. (2017) 27:297–301. 10.1016/j.annepidem.2017.05.002 28595734

[22] Knoppers BM Thorogood A Ethics and big data in Health. Curr Opin Syst Biol. (2017) 4:53–7. 10.1016/j.coisb.2017.07.001

[23] Rodrigues JJ de la Torre I Fernandez G Lopez-Coronado M. Analysis of the security and privacy requirements of cloud-based electronic health records systems. J Med Internet Res. (2013) 15:e186. 10.2196/jmir.2494 23965254 PMC3757992

[24] Harvey C Big Data Management Datamation Daily Newsletter. (2017). Availableonline at: https://www.datamation.com/big-data/big-data-management.html (accessed June 20, 2017).

[25] Barrot CC Woillard JB Picard N. Big data in pharmacogenomics: current applications, perspectives and pitfalls. Pharmacogenomics. (2019) 20:609–20. 10.2217/pgs-2018-0184 31190620

[26] Assale M Dui LG Cina A Seveso A Cabitza F. the revival of the notes field: leveraging the unstructured content in electronic health records. Front Med. (2019) 6:66. 10.3389/fmed.2019.00066 31058150 PMC6478793

[27] He KY Ge D He MM. Big data analytics for genomic medicine. Int J Mol Sci. (2017) 18:E412. 10.3390/ijms18020412 28212287 PMC5343946

[28] Murdoch TB Detsky AS. The inevitable application of big data to health care. JAMA. (2013) 309:1351–2. 10.1001/jama.2013.393 23549579

[29] Hughey JJ Rhoades SD Fu DY Bastarache L Denny JC Chen Q. Cox regression increases power to detect genotype-phenotype associations in genomic studies using the electronic health record. BMC Genomics. (2019) 20:805. 10.1186/s12864-019-6192-1 31684865 PMC6829851

[30] Lee J Hamideh D Nebeker C. Qualifying and quantifying the precision medicine rhetoric. BMC Genomics. (2019) 20:868. 10.1186/s12864-019-6242-8 31730456 PMC6858780

[31] Stuart D Allin K Penny D Lucraft M Astell M Practical Challenges for Researchers in Data Sharing. Springer Nature; Springer; NatureResearch; BMC; Palgrave MacMillan (2018). 10.6084/m9.figshare.5971387. (accessed March 21, 2018).

[32] Raza S Hall A. Genomic medicine and data sharing. Br Med Bull. (2017) 123:35–45. 10.1093/bmb/ldx024 28910995 PMC5862236

[33] Wilkinson MD Dumontier M Aalbersberg IJ Appleton G Axton M Baak A. The FAIR Guiding Principles for scientific data management and stewardship. Sci Data. (2016) 3:160018. 10.1038/sdata.2016.18 26978244 PMC4792175

[34] Shabani M Dove ES Murtagh M Knoppers BM Borry P. Oversight of genomic data sharing: what roles for ethics and data access committees? Biopreserv Biobank. (2017) 15:469–74. 10.1089/bio.2017.0045 28836815

[35] Trepanier A Ahrens M McKinnon W Peters J Stopfer J Grumet SC. Genetic cancer risk assessment and counseling: recommendations of the national society of genetic counselors. J Genet Couns. (2004) 13:83–114. 10.1023/B:JOGC.0000018821.48330.77 15604628

[36] Trepanier A Ahrens M McKinnon W Peters J Stopfer J Grumet SC Cancer Genetics Risk Assessment and Counseling (PDQ®)–Health Professional Version. National Cancer Institute PDQ Available online at: https://www.cancer.gov/about-cancer/causes-prevention/genetics/risk-assessment-pdq#_1004 (accessed March 1, 2019).

[37] The SHARE Approach. The SHARE Approach. (2014). Available online at: https://www.ahrq.gov/health-literacy/curriculum-tools/shareddecisionmaking/index.html (accessed August 2018).

[38] U.S. PSTF BRCA-Related Cancer: Risk Assessment, Genetic Counseling, and Genetic Testing USPSTF Website. (2019). Available online at: https://www.uspreventiveservicestaskforce.org/Page/Document/UpdateSummaryFinal/brca-relatedcancer-risk-assessment-genetic-counseling-andgenetic-testing1?ds=1&s=BRCA-related%20cancer (accessed February 19, 2020).

[39] National Comprehensive Cancer Network IU. Genetic/Familial High-Risk Assessment. Available online at: https://www.nccn.org/professionals/physician_gls/pdf/genetics_screening.pdf

[40] Ashley EA Butte AJ Wheeler MT Chen R Klein TE Dewey FE. Clinical assessment incorporating a personal genome. Lancet. (2010) 375:1525–35. 10.1016/S0140-6736(10)60452-7 20435227 PMC2937184

